# Dosimetric Validation of a System to Treat Moving Tumors Using Scanned Ion Beams That Are Synchronized With Anatomical Motion

**DOI:** 10.3389/fonc.2021.712126

**Published:** 2021-09-08

**Authors:** Michelle Lis, Wayne Newhauser, Marco Donetti, Moritz Wolf, Timo Steinsberger, Athena Paz, Christian Graeff

**Affiliations:** ^1^Biophysics, GSI Helmholtzzentrum für Schwerionenforschung GmbH, Darmstadt, Germany; ^2^Department of Physics and Astronomy, Louisiana State University, Baton Rouge, LA, United States; ^3^Department of Radiation Physics, Mary Bird Perkins Cancer Center, Baton Rouge, LA, United States; ^4^Research and Development Department, CNAO National Center for Oncological Hadrontherapy, Pavia, Italy; ^5^Institute of Condensed Matter Physics, Technical University of Darmstadt, Darmstadt, Germany

**Keywords:** motion-synchronized dose delivery, carbon ion therapy, range compensation, motion mitigation, multi-phase 4D delivery, scanned ion beams

## Abstract

**Purpose:**

The purpose of this study was to validate the dosimetric performance of scanned ion beam deliveries with motion-synchronization to heterogenous targets.

**Methods:**

A 4D library of treatment plans, comprised of up to 10 3D sub-plans, was created with robust and conventional 4D optimization methods. Each sub-plan corresponded to one phase of periodic target motion. The plan libraries were delivered to a test phantom, comprising plastic slabs, dosimeters, and heterogenous phantoms. This phantom emulated range changes that occur when treating moving tumors. Similar treatment plans, but without motion synchronization, were also delivered to a test phantom with a stationary target and to a moving target; these were used to assess how the target motion degrades the quality of dose distributions and the extent to which motion synchronization can improve dosimetric quality. The accuracy of calculated dose distributions was verified by comparison with corresponding measurements. Comparisons utilized the gamma index analysis method. Plan quality was assessed based on conformity, dose coverage, overdose, and homogeneity values, each extracted from calculated dose distributions.

**Results:**

High pass rates for the gamma index analysis confirmed that the methods used to calculate and reconstruct dose distributions were sufficiently accurate for the purposes of this study. Calculated and reconstructed dose distributions revealed that the motion-synchronized and static deliveries exhibited similar quality in terms of dose coverage, overdose, and homogeneity for all deliveries considered. Motion-synchronization substantially improved conformity in deliveries with moving targets. Importantly, measurements at multiple locations within the target also confirmed that the motion-synchronized delivery system satisfactorily compensated for changes in beam range caused by the phantom motion. Specifically, the overall planning and delivery approach achieved the desired dose distribution by avoiding range undershoots and overshoots caused by tumor motion.

**Conclusions:**

We validated a dose delivery system that synchronizes the movement of the ion beam to that of a moving target in a test phantom. Measured and calculated dose distributions revealed that this system satisfactorily compensated for target motion in the presence of beam range changes due to target motion. The implication of this finding is that the prototype system is suitable for additional preclinical research studies, such as irregular anatomic motion.

## Introduction

Proton and ion therapy provide conformal dose distributions for static targets, and in the past few decades, have emerged as a formidable alternative to photon therapy. Ion therapies have mainly been used to treat static tumors, including several in the regions of the head and neck region, cranium, retina, and the spine, with high conformity (1, 2), resulting in reduced toxicities and tumor recurrence (3). Conformal treatments have been shown to be partially effective in reducing complications associated with radiotherapy of moving tumors, such as non-small cell lung cancer, including pneumonitis and cardiac complications (4, 5). However, contemporary ion beam therapies for thoracic tumors still have high complication rates and low survival rates (6). Additional unmet clinical needs include shorter treatment times and streamlined patient-specific quality assurance procedures. Thus, it is imperative to develop treatment methods that can meet these clinical needs.

Currently, about two thirds of proton and ion therapy centers use relatively simple motion mitigation strategies to treat moving tumors, including various combinations of techniques such as breath hold, beam gating, and internal target volumes (ITV), used with or without rescanning (7). These motion mitigation strategies for scanned ion therapy have been used to successfully treat some, but not all, moving tumors, yet treatment complication rates remain a serious problem (8, 9). The local failures are largely believed to be caused by insufficient dose to the tumor and complications are believed to be caused by excessive dose to surrounding healthy tissues (10). An obvious approach to overcome these limitations is to amend treatment planning and dose delivery methods to increase tumor coverage and reduce dose to normal tissues. To achieve these, improvements are needed to mitigate against range variations that are induced by moving heterogenous anatomy, including cases where the movements of the tumor and surrounding healthy tissue differ from one another. The most advanced motion mitigation approach currently in clinical use, namely the phase-controlled rescanning method at the National Institute of Radiological Sciences (NIRS), combines rapid beam delivery with fluoroscopy-guided beam gating. This requires minimal changes to the target position during the time where the treatment unit actively delivers beam to the tumor. With this approach, treatments must be halted if tumor motion changes substantially from the expected tumor location (11). The advantage of the gating approach is that it avoids the technical complexity of motion mitigation, but increases the compliance requirements of patients, and some patients cannot comply with respiratory requirements. The most technologically advanced approach, commonly called 4D-optimized tracking, allows the patient to breath freely and requires the treatment system to modify the trajectories of the delivered ion beams to follow the moving tumor, using real-time monitoring of the tumor position. This approach, developed at GSI for more than a decade (12), revealed promising dosimetric qualities and technical feasibility, but the vast technological complexity required to compensate anatomical motion has thus far been a major obstacle to its translation to clinical practice. To overcome these obstacles, Lis et al. (13) developed a technologically straightforward approach, called multi-phase 4D beam delivery (MP4D), which provides comparable dosimetric quality to that of beam tracking without the associated complications. It takes anatomical and tumor motion into account during treatment planning and subsequently synchronizes the beam delivery in real-time so that it follows the moving tumor. The MP4D approach was previously characterized for moving targets with promising preliminary results, but the tests did not attempt to compensate for range changes that occur in a heterogeneous phantom. To our knowledge, no system with such capabilities has yet been validated or clinically commissioned.

The objective of this study was to validate, by measurement and calculation, the performance of a recently created motion-synchronized dose delivery system (M-DDS) (13), used to deliver MP4D treatments. In particular, we validated, for the first time, the ability of the M-DDS to compensate for tumor motion in the presence of anatomical, motion-induced range changes. Libraries of 4D-optimized carbon-ion treatment plans were delivered to phantoms and absorbed dose distributions were measured. The dosimetric quality was assessed by examination of the dose coverage, conformity, overdose and uniformity. These quantities were compared for deliveries with a variety of test cases, including those with stationary and moving tumors, with and without the application of motion synchronization.

## Materials and Methods

We validated a prototype system to treat moving targets with scanned ion beams. The overall approach was to synchronize the delivery of the beam to the periodic motion of the target, to allow for almost continuous delivery of the beam to the moving target. This approach inherently includes compensating for motion in heterogenous anatomy, which would otherwise cause range over- and under-shoots due to the anatomical, motion-induced range changes. For the convenience of the reader, we briefly review here the previously reported methods for motion mitigation with MP4D deliveries (13, 14), the treatment planning system (TPS) (11), and the experimental apparatus (13). We then describe the analysis methods for assessing the impact of managing heterogenous anatomical motion with the multi-phase 4D approach.

### Treatment Planning System and Treatment Delivery System

Treatment plans were created with the research TPS developed at GSI Helmholtzzentrum für Schwerionenforschung GmbH (GSI), called TRiP4D (15). This is an extension of TRiP98 (16, 17), which takes into account changes in patient anatomy caused by respiratory motion. Several previously established planning strategies were used, including conventional- and robust 4D optimization and conventional- and robust 3D optimization (these are each explained below). To create a 4D treatment plan, a 3D sub-plan is created on each of the respiratory motion phases found in a 4DCT image set. The library of sub-plans is utilized together as a complete, or composite, treatment plan. In this study, we used two simple phantoms to represent the human thorax and a moving tumor. We purposefully selected simple phantoms to facilitate direct comparisons of calculated and measured dose distributions. These comparisons were essential for validating the dosimetric performance in test scenarios where the target depth or range varied in time. More specifically, two types of variations were considered, including constant range variations (created with a moving, homogenous wedge), and discrete range variations (created by a moving slab containing heterogeneities).

4D treatment plans were created for each phantom. First, 4DCT image sets were created by shifting a 4/3π 3 × 3 × 2 cm^3^ ellipsoid target or a 6 × 6 × 4 cm^3^ cuboid target contour within a simulated water box phantom. The targets followed a 20 mm, Lujan2-type motion trajectory (18) that was lateral to the beam axis. To explore the impact of the number of motion phases on delivery quality, we created 4D plans containing 3, 6 and 10 motion phases. Sub-plans were optimized to cover the clinical target volume (CTV) in each motion phase with a fraction of the prescription dose, such that the sum of the sub-plan doses results in the target receiving the prescription dose. For 3D optimization, 3DCT image sets of the ellipsoid and cuboid targets were created and used during treatment planning.

Analogous treatment plans were created using conventional and robust optimization planning techniques. For conventional optimization, treatment plans were created for CTVs which had 3 mm isotropic margins, while for robust optimization, margins were calculated from nine uncertainty scenarios, including range uncertainties and target position shifts, to minimize their dosimetric impact. Robust optimization was described by Wolf et al. (19). Robustly and conventionally optimized plan libraries were created for both target volumes, on all 4DCT images and to a homogeneous absorbed dose of 3 Gy.

The plan libraries were delivered with the motion-synchronized dose delivery system (M-DDS) (13), which was created to accelerate research and translation of motion mitigation strategies in ion therapy. This system was implemented in a research version of the dose delivery system (DDS) that is used clinically at the National Center for Oncological Hadrontherapy (CNAO) (20). It was similarly implemented in the radiotherapy research facility (Cave M) at GSI (21).

The general approach considers motion of the entire anatomy during treatment planning. This allows for compensating for the motion of heterogeneous tissue and variable target depths without the need for real-time modifications to the beam spot delivery positions during delivery. Instead, the real-time target position is monitored to redirect delivery from sub-plan to sub-plan, as the target moves to another motion phase. As such, the sub-plans are delivered as a series of discrete stationary plans. This continues until the entire prescription dose is delivered. For this study, up to 10 motion phases were considered, corresponding to the number of phases typically found in a 4DCT for lung cancer patients; however, additional motion phases can be trivially added if needed. Though the number of motion phases is discrete, the tumor motion is continuously monitored, and a variety of motion monitoring devices can be selected.

In this study, we used continuous monitoring of target motion to adapt the delivery sequence of sub-plans. The 1D target motion was monitored with an optical distance sensor (OD100—35P840, SICK, Waldkirch, Germany). The signal amplitude was digitized and analyzed to yield a discrete motion phase. The sub-plan found in the plan library, corresponding to the detected phase, was then accessed. During beam delivery, the beam spots in the sub-plan that corresponded to the detected motion phase were delivered in sequence until complete, or until another motion phase was detected. When another phase was detected, the delivery was then redirected to the nearest beam spot within the corresponding sub-plan and delivery continued as before. Once all of the spots in an iso-energy slice (IES) were delivered for the given sub-plan, the beam was suspended until delivery was directed to a sub-plan containing yet undelivered spots of the same energy. This process continued until all beam spots for that IES were delivered, then delivery progressed to the next IES. For deliveries to static targets (plan libraries with one motion phase), all the beam spots are delivered in sequence for each IES until all beam spots were delivered.

At the time of this study, the refurbished accelerator system at GSI was only capable of single-energy deliveries and the beam range was modulated with a range shifter to deliver beams to entire target volumes. Subsequent work will implement fast and automated switching between accelerated beam energies to efficiently deliver multiple beam energies. Therefore, all plans were delivered with a nominal beam energy of 280 MeV/u. As a provisional means to produce multiple beam energies and ranges within a single delivery, we used a binary range shifter comprising polyethylene (PE) plastic slabs (21, 22). The treatment plans contained beam energy codes, that specified the needed beam energy for each slice of the treatment plan. These codes were converted to range shifter settings, where the range shifter settings specified the insertion of a combination of range shifting absorber slabs to modulate the beam range. Each of the selected binary codes corresponds to a combination of plastic slabs that allowed for shifting the beam spots longitudinally by as little as 0.1 mm increments. During beam delivery, the switching of range shifter settings was synchronized with the spill cycles of the synchrotron. The range shifter was further described elsewhere (22).

This motion-synchronized dose delivery system was previously implemented into the M-DDS and preliminary tests were reported (13).

### Experimental Setup

**Figure 1:**
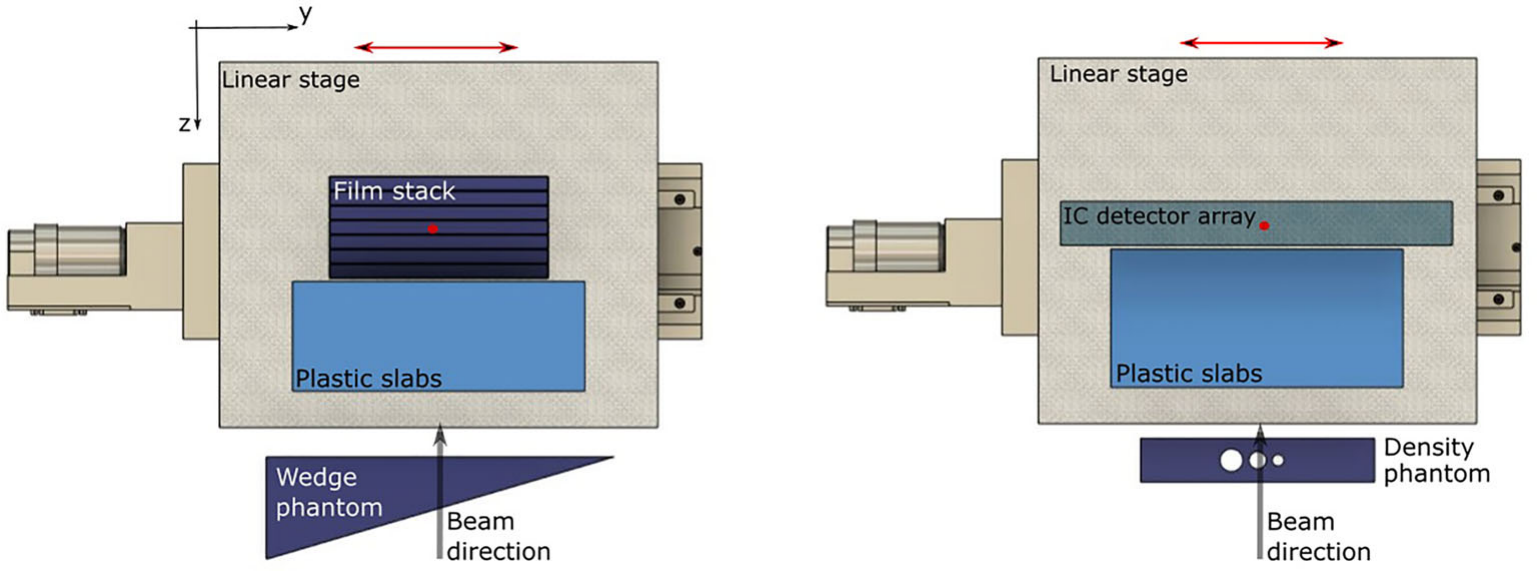
Setups for testing motion compensation through heterogenous targets. A combination of four setups were used with either the wedge or density phantom and where measurements were made with either an IC array detector or film stack. A top view of the wedge-shaped phantom (left) and slab phantom with density heterogeneities (right) are shown, placed in front of a periodically moving linear stage. For all setups, the dosimeter was placed behind a set of plastic slabs. Isocenter is marked with a red circle and the linear stage movement is indicated with a red arrow.

Plan libraries were delivered to two setups, containing a heterogenous phantom, and moving slabs and dosimeters (**Figure 1**). Treatment deliveries were repeated twice for each setup: once to irradiate a 2D ionization chamber (IC) array detector (Octavius 1500XDR; PTW, Freiburg, Germany) and again to a stack of six radiochromic films (EBT3 Gafchromic; International Specialty Products, Wayne, NJ). The 2D IC array detector was placed within a 5 mm thick PMMA holder, and 6 films, were slotted into the films stack, between 10 mm slabs of PMMA (21). The 2D IC array detector, containing ICs filled with air at ambient pressure, was set to integral mode to measure total delivered dose. Both holders were mounted on top of a motorized linear stage (M-414.2PD; Physik Instrumente (PI) GmbH, Karlsruhe, Germany), aligned perpendicularly to the beamline. Slabs of water equivalent plastic, corresponding to 56.7 mm water-equivalent thickness (WET) were placed in front of these holders, on the linear stage. The linear stage was programmed to move with a 20 mm, uni-axial Lujan2-type respiratory motion-like pattern (18), and the motion was monitored in real time with an optical distance laser sensor (13, 23).

Both setups contained a range modifying phantom. These included a wedge-shaped piece of PMMA (‘wedge phantom’) and a rectangular shaped piece of PMMA (‘density phantom’) with three air cavities (**Figure 1**). These were used to test uniform, gradual changes to the thickness proximal to the target, and discrete gradients from air gaps, respectively. The wedge phantom was 100 × 70 × 128 mm^3^, with a lateral slope of 0.3 mm/mm. The density phantom was a 160 × 109.9 × 30 mm^3^ block of PMMA with 15.8-, 12.0-, and 8.1-mm diameter cylindrical air gaps. In both cases, the wedge and density phantoms remained stationary and were placed in front of the detector and water-equivalent plastic slabs, which were placed on top of the moving linear stage. The density phantom induced range changes of 5.5 to 18.4 mm and the wedge phantom induced a maximum range gradient of 8.8 mm/mm. In addition to the MP4D deliveries, 3D optimized plans were delivered to moving targets, without motion mitigation, to assess the dosimetric impact of motion interplay effects. Similarly, 3D-optimized plans were delivered to static targets to determine the reference dosimetric performance of the treatment delivery system.

### Data Analysis

The dosimetric quality of deliveries through the multiple range phantoms was analyzed by reconstructing beam monitoring data from treatment delivery log files of the M-DDS. The dose delivery data log files were reconstructed on the 4DCT images containing a simulated water-box phantom. The dose delivery data log files and motion monitoring data log files, from the motion monitoring system (13), were parsed and reformatted into the TRiP4D treatment plan format. TRiP4D was then used to calculate (reconstruct) the delivered dose distributions from the reformatted files. The dose distributions were calculated on the same target volumes as used during treatment planning. We then compared the reconstructed, planned and measured dose distributions. The dosimetric quality metrics we selected to assess motion management included uniformity, conformity, dose coverage and overdose. Each of these metrics were calculated from dose volume histogram (DVH) data from planned and delivered dose reconstructions. Conformity number is

(1) $$CN=\frac{V_{T,p}}{V_{T}}\times \frac{V_{T,p}}{V_{p}}$$

where *V_T,p_* is the portion of the CTV that receives a dose that is greater than or equal to the prescribed dose, *D_p_*, *V_T_* is the CTV, and *V_p_* is the volume that receives a dose that is greater than or equal to *D_p_* (24). A CN value of 100% is ideal and, while there is no threshold for an acceptable CN, we considered > 60% to be acceptable. Homogeneity (HI), which is a measure of delivery uniformity, is

(2) $$HI=D_{5}-D_{95}$$

where *D_5_* and *D_95_* are the percentages of the prescription dose, *D_p_* = 3 Gy, which are delivered to 5 % and 95 % of the tumor volume, respectively (25). An HI of 0 % is ideal and < 5 % was considered acceptable (26). Tumor dose coverage, which is the percentage volume of the CTV that received at least 95% of the *D_p_*, is represented by *V_95_*. A *V_95_* of 95% was considered clinically acceptable. Overdose, denoted by *V_107_*, is the percentage of the CTV that receives over 107 % of the prescription dose. Zero overdose is ideal. The acceptable ranges for these metrics were selected for the purposes of this study.

Each measured dose distribution was compared to the corresponding planned dose distribution and reconstructed dose distribution. The 3D generalized gamma index analysis (27), was used to quantify the degree of agreement between each pair of dose distributions. With the generalized gamma index analysis, we were able to objectively select magnitudes for the distance to agreement and dose difference criteria for our data set. This allowed for more accurate characterization of the dosimetric agreement in the low-dose region. Pass criteria of 3 % dose difference and 3 mm distance to agreement were applied in all cases. Pass rates of > 90 % were considered acceptable. The purpose of comparing measured and reconstructed dose distributions was to validate the accuracy of the dose reconstruction method. The purpose of comparing measured and planned dose distributions was to determine the amount of unintended delivered dose during beam gating and spill pauses.

### Quality Assurance

We performed limited quality assurance (QA) on the beam output prior to dosimetry measurements. The QA comprised relative dosimetry, using the methods described by Luoni et al. (28). Specifically, the constancy of the beam output (relative absorbed dose) was measured with a farmer-type ionization chamber (PTW 30010 Farmer Chamber; PTW, Freiburg, Germany), placed at isocenter. The farmer chamber was inserted into a 30 × 30 × 1 cm^3^ water-equivalent plastic holder slab, with a water-equivalent point of measurement at 5 mm depth. A 5 × 5 cm^2^ square field of 2 Gy absorbed dose was delivered with 280 MeV/u carbon ion beams without range modulation. Each measurement was repeated three times. The same field was delivered to a film at 5 mm depth to measure field homogeneity. This QA approach was selected because it is well established, fast and because beamtime for QA procedures was severely limited at the time of this study.

We defined reference conditions to facilitate calibrations of two dosimeters (a 2D IC array detector and radiochromic films). The reference conditions comprise three major elements, namely, a reference radiation field, a reference phantom, and a reference measurement location. The reference radiation field comprised a 280 MeV/u carbon ion beam without range modulation and with an incident beam spot size of 6.7 mm full-width half maximum, delivered to a measurement depth of 5 mm, at isocenter.

We calibrated the 2D IC array detector and film to absorbed dose under reference conditions. The calibrations of these dosimeters were based on measurements of their responses to irradiations of known absorbed dose. The known absorbed dose was determined from dose reconstructions, which were previously calibrated and are described elsewhere (11). We simultaneously calibrated the IC array detector and films. The detector was positioned at isocenter, inside of a 31 × 40 × 4 cm^3^ box-like holder with 5 mm water-equivalent thick walls. A film was taped directly in front of the 2D IC array detector, inside the holder. A calibration plan was delivered that comprised eight 30 × 30 mm^2^ square fields, ranging in fluences from 5 × 10^4^ to 1 × 10^7^ particles/mm^2^, corresponding to absorbed doses of 0.1 to 9.9 Gy at 1 cm depth, in the plateau region. The initial beam energy was 280 MeV/u, and no additional range modulation was introduced. This interval of absorbed dose values was selected to encompass the dynamic range anticipated for the clinical deliveries and to remain within the dynamic range of optical densities for radiochromic films.

We used an established formalism to calibrate the IC array detector (13, 21, 29). These are briefly reviewed here for the convenience of the reader. The IC array detector was calibrated to absorbed dose to water under reference conditions. Measured absorbed dose is given by

(3) $$D_{IC}=M\times C\times k_{Q}$$

where *M* is the measured response (corrected for leakage, temperature, and pressure) and *C* is the calibration coefficient under reference conditions, *k_Q_* corrects for the effects of the difference between the reference conditions and the non-reference conditions. We confirmed the stability of the previously determined value of *C* = 1.2 Gy per unit of measured response, following methods similar to those described by Stelljes et al. (30). The effects of non-reference conditions were negligible and *k_Q_* was approximated as unit value. The absolute absorbed dose values, at the same locations as the ICs and under reference conditions, were also reconstructed from delivery log files, which allowed us to calculate dosimetric outcomes from the reconstructions.

We used radiochromic films to simultaneously measure relative 2D absorbed dose distributions under reference and non-reference conditions. Films allowed for faster data acquisition at multiple depths during the limited beamtime available. They also provided the high spatial resolution needed to measure dose distributions distal to the wedge and density phantoms.

We used methods similar to those of Yonai et al. (31) for calibrating the film response to relative absorbed dose, which are briefly reviewed here for the convenience of the reader. First, the TPS was used to create a calibration plan under reference conditions, described above. The calibration plan was delivered to the radiochromic film, in the geometry described above. The exposed films aged for one day, then were digitized (DosimetryPro Advantage Red; VIDAR Systems Corporation, Herndon, VA, USA) using a 16-bit sampling and 300 dots per inch resolution. The net optical density of the scanned film was determined by

(4) $$OD_{net}=OD_{m}-OD_{bkg}$$

where *OD_m_* is the measured (scanned) optical density, and *OD_bkg_* is the background optical density scanned in an unirradiated area on each film. The *OD_net_* was determined in the central region of each square field in the calibration film. The known absorbed dose values at the center of each square field, *D_film_*, were fit to eight measured *OD_net_* values according to

(5) $$D_{film}=D_{film,uncorr}\times k_{Q,film}$$

where *D_film,uncorr_* is the uncorrected, measured absorbed dose from films, and *k_Q,film_* is a correction factor for changes in the film response due to changes in beam quality at non-reference conditions, including other depths. The value *k_Q,film_* corresponds to a factor that is called relative efficiency elsewhere (31, 32). By definition, *k_Q,film_* took a value of 1 at the reference condition used for the calibration. Under non-reference conditions, the value of the correction factor *k_Q,film_* corrected for changes in the film response due to quenching, which depends on beam quality, as specified by the beam’s linear energy transfer. Both *D_film,uncorr_* and *k_Q,film_* were calculated using methods modified from Yonai et al. (31). The calibration procedure above was performed separately for each batch of film used.

## Results

**Figure 2:**
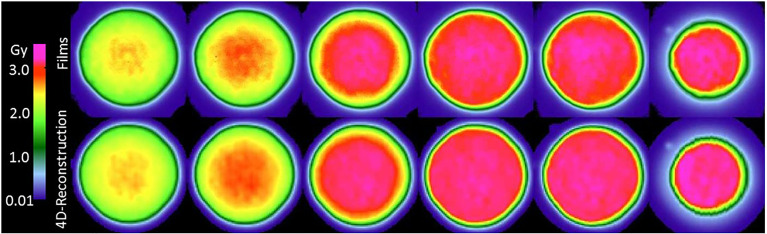
Comparison of film measurements (top row) to dose reconstructions (middle row). The top row shows the films from a film stack for a multi-phase 4D delivery using 10 motion phases to the wedge phantom with 20 mm uniaxial motion. Films are ordered left to right with increasing depth in the wedge phantom. The depth increment between films is approximately 11 mm water-equivalent thickness. The corresponding dose reconstructions for the same delivery are shown in the middle row.

**Figure 2** reveals that the reconstructed dose distributions agree well with the corresponding dose distributions obtained from measurements with film. This result confirms the suitability of the method for reconstructing dose distribution for the main purpose of this study, which is to assess the quality of dose distributions delivered by various techniques. We defer discussion of the results on confirming of the reconstruction methods until later in this section.

### Dosimetric Validation

Relative dosimetry was performed prior to experiments by delivering a uniform square profile to a farmer-type IC and a radiochromic film. Output constancy was verified, and beam output ranged from 2.57 to 2.59 Gy at isocenter. Field homogeneity was also measured with the homogeneity index, and was 4.8%.

**Figure 3:**
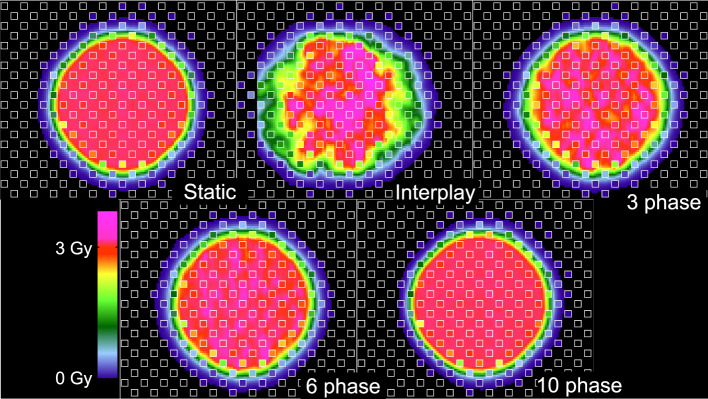
Comparison of 2D ionization chamber array detector measurements to dose reconstructions. The bottom row shows measured absorbed dose values (values inside of small white squares) overlaid on reconstructed absorbed dose distributions (values outside of the small white squares). The dose distributions are distal to the density phantom (see **Figure 1**). Distributions are from four delivery techniques: static target, moving target without motion compensation (interplay), and moving target with multi-phase 4D motion compensation. Multi-phase 4D deliveries are shown using 3, 6, and 10 motion phases in the treatment plan libraries.

We assessed the dosimetric quality of deliveries through the wedge and the density phantoms. Measured absorbed dose distributions were compared to the corresponding dose distributions from reconstructions and treatment plans. **Figure 3** plots the absorbed dose distributions for these deliveries, including those with a static target, moving target without motion compensation (revealing the extent of interplay), and moving target with the multi-phase 4D approach (revealing the effectiveness of motion mitigation).

**Figure 4:**
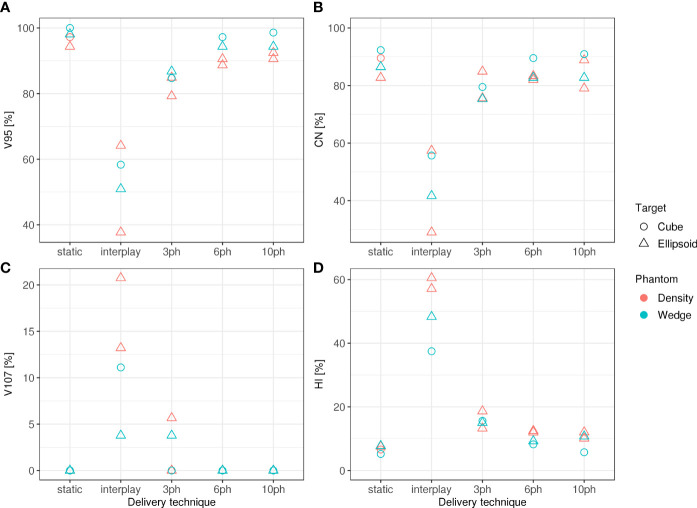
**(A)** Dose coverage (V_95_), **(B)** conformity (CN), **(C)** overdose (V_107_), and **(D)** homogeneity (HI) for static deliveries to stationary targets (static), static deliveries to moving targets (interplay), and 3-phase, 6-phase and 10-phase multi-phase 4D deliveries to cube and ellipsoid target volumes through the wedge and density phantom.

**Figure 5:**
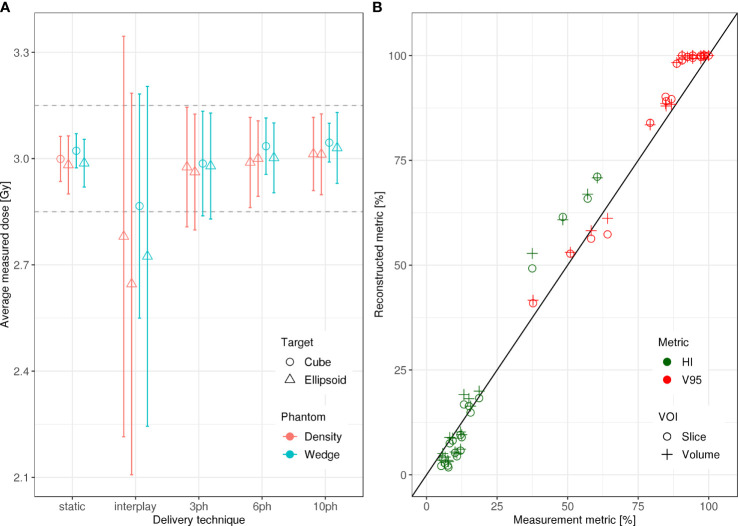
**(A)** Average measured absorbed dose *versus* delivery technique evaluated using the density and wedge phantoms. The dashed grey lines delineate the ± 5 % tolerance interval centered about the prescription absorbed dose of 3 Gy and the bars indicate the spread measured dose values from each ionization chamber (IC) of the 2D IC array detector in the clinical target volume (CTV) **(B)** Homogeneity (HI) and coverage (V_95_) for measured *versus* reconstructed absorbed dose distributions in the target volume. Dosimetric quality was calculated in a single iso-energy slice within the CTV and in the entire CTV for each delivery. All delivery techniques for both phantom types and for both VOIs are plotted. Data points that fall on the line indicate full agreement of measured and reconstructed HI and V_95_ values.

Specifically, dosimetric quality was assessed with four metrics: conformity, homogeneity, coverage, and overdose (**Figure 4**). The major qualitative finding from these results is that 10-phase MP4D deliveries provided the best overall dosimetric quality. The major qualitative finding is that 10-phase and 6-phase MP4D deliveries had acceptable dosimetric quality, while quality metrics for 3-phase MP4D were mixed. **Figures 2**, **3** reveals that the reconstructed dose distributions agree well with measurements. Acceptable conformity (*CN* > 60 %) was obtained in all MP4D deliveries with 6 and 10 phases. However, at least 10 motion phases were required to achieve acceptable homogeneity (*HI* < 5 %) of the absorbed dose in the CTV. Fewer motion phases produced unacceptably large heterogeneities, due to interplay effects within each motion phase (so-called “residual motion”). The average *HI* value for all of the 10-phase MP4D deliveries (both phantom types and CTV shapes) was 8 %, approaching the criteria of < 5 %, which was achieved for static deliveries and is considered acceptable for other deliveries. Target coverage was 100 % for 10-phase MP4D deliveries and was > 98 % for 6-phase MP4D deliveries, also approaching the ideal results of 100%, which were obtained from deliveries to a static target. These findings on coverage and heterogeneity are qualitatively supported by dose distributions plotted in **Figure 3**, which shows that the MP4D approach produces similar results for the static and 10-phase MP4D deliveries. It was expected that the deliveries using 10 motion phases would have superior results, since the residual motion was less than that with 6 or 3 motion phases. The increasing homogeneity is also seen in **Figure 5**, where the range of average measured absorbed dose values narrow with increasing number of motion phases. Here, the average absorbed dose was within ± 1.5 % of the prescription dose for MP4D deliveries. Finally, the 10-phase MP4D approach produced hotspots in the CTV that were < 103% of the prescribed absorbed dose. Together, these findings suggest that the 10-phase MP4D approach provides good dosimetric quality that closely approaches the quality that was achieved for static-target deliveries.

### Effectiveness of Multi-Phase 4D Delivery

**Figure 6:**
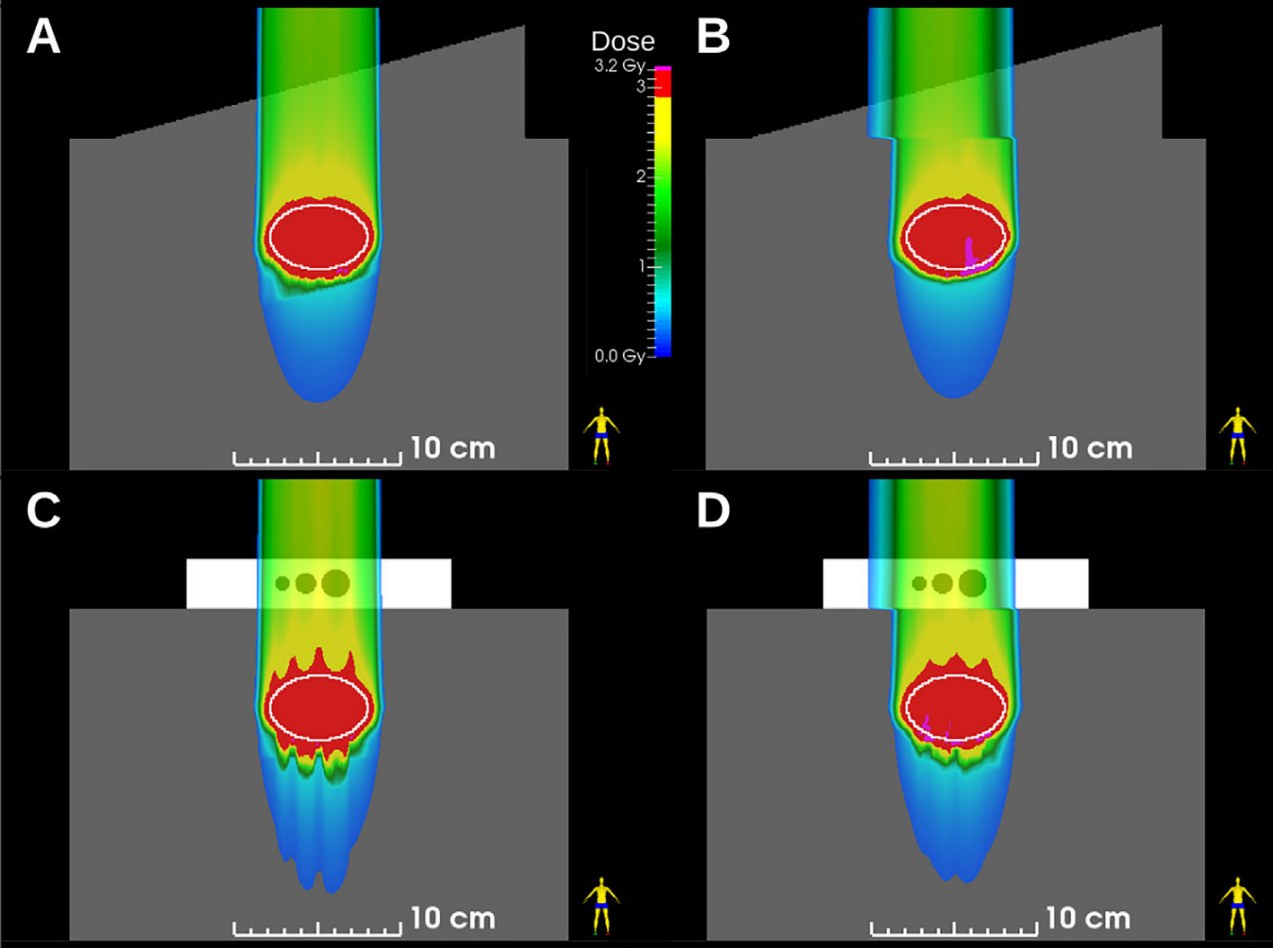
Dose distributions in ellipsoid targets (white ovals) for **(A)** static and **(B)** 10-phase multi-phase dose deliveries through the wedge phantom and for **(C)** static and **(D)** 10-phase multi-phase 4D deliveries through the density phantom. Plans were robustly optimized and deliveries were reconstructed from beam delivery log files and motion monitoring log files.

The results of MP4D deliveries also yielded important findings regarding over- and undershoot of the beam range and regarding the inverse interplay effect. Regarding range effects, absorbed dose distributions from treatment plans and dose reconstructions are shown in **Figure 6** (SlicerRT, Kingston CA), which illustrates that static robust optimization created dose distributions with range over- and undershoots. These are a consequence of taking the large range uncertainties in low density material of the phantom into account. These range effects manifest distal to the cavities of the density phantom, near the end of range. The MP4D approach reduced these range defects as, the dose delivered to these regions were “blurred out” by delivering multiple sub-plans to the target volumes. Regarding the inverse interplay effect, the MP4D approach exhibited no dose defects from this (**Figures 6B, D**). The inverse interplay effect is a serious concern that is associated with the beam tracking delivery approach (15). With beam tracking, the beam spot positions are modified from their planned positions to compensate for detected real-time motion. This can deliver uniform doses to the target but introduces hotspots and cold spots in the beam path, proximal to the target in healthy tissue. With the MP4D approach, there were no hotspots in the proximal healthy tissue. Instead, the lateral extent of the irradiated healthy tissue was broadened by the amplitude of the target motion. These findings on range defects and inverse interplay further suggest that MP4D approach can provide high quality deliveries.

**Figure 7:**
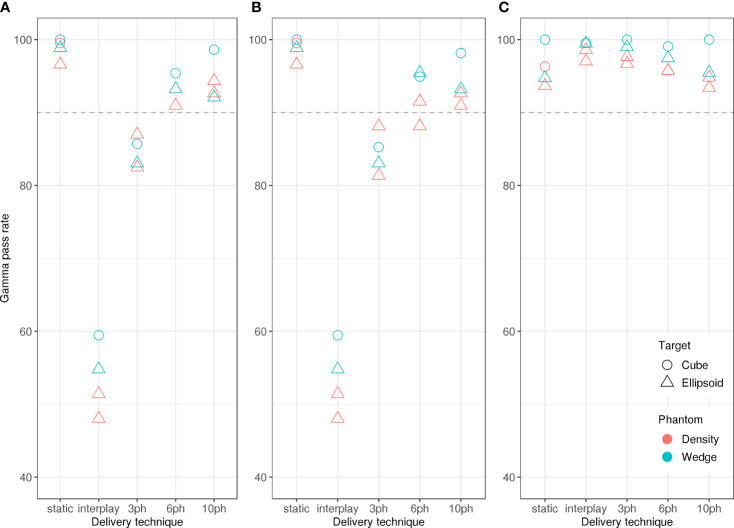
Gamma index analysis pass rates *versus* beam delivery technique. The pass rates indicate good agreement between measured absorbed dose distributions and **(A)** reconstructed absorbed dose distributions for the static delivery, **(B)** planned absorbed dose distributions and **(C)** reconstructed absorbed dose distributions. Comparisons were made for static deliveries to stationary targets (static), static deliveries to moving targets (interplay), and multi-phase deliveries to moving targets with 3 phases, 6 phases, and 10 phases of motion compensation. Pass rates showed only a weak dependence on phantom type (wedge or density types) and target shape (cube or ellipsoid types).

**Figure 2** reveals that the dose reconstruction methods were confirmed by measurements. In particular, high gamma-index pass rates confirmed the accuracy of the treatment planning and dose reconstructions in this study. Specifically, we compared dose distributions from measurements with the IC detector array to those from the corresponding log file reconstructions (**Figure 7C**) and treatment plans (**Figure 7B**) obtained with moving targets. Average pass rates increased with the number of motion phases, due to the decreasing residual motion within each motion phase. In all cases, reconstructed dose distributions agreed well with measured dose distributions (**Figure 7C**), with pass rates > 90 %, confirming the validity of the dose reconstruction strategy. Gamma index analysis pass rates were lower for comparisons between planned and measured dose distributions and only static deliveries and 10-phase MP4D deliveries had pass rates > 90 %.

Similarly, we compared all dose distributions from measurements to reconstructions of static deliveries (**Figure 7A**). This comparison provided important contextual information on the magnitude of dose degradations that were caused by target motion occurring within a motion phase and without motion compensation. Pass rates were < 90 % for comparisons between static reconstructions and 3 phase MP4D measurements and were < 60 % for comparisons between static reconstructions and interplay deliveries. For planning studies, 10 or more motion phases should be selected.

The total delivery time was calculated from treatment log files. Average total delivery time for static ellipsoid deliveries was 7.4 min. The relative increase in delivery time for 3-phase, 6-phase and 10-phase MP4D deliveries, compared to static deliveries, was 7%, 17%, and 21%, respectively. This suggests that the MP4D method provides motion-mitigated deliveries with increases to delivery time that would be well-tolerated by most radiotherapy patients and compatible to existing patient caseloads.

## Discussion

In this study, we validated the dosimetric performance of a novel multi-phase 4D treatment approach with deliveries to heterogenous phantoms. Specifically, we measured dosimetric quality of absorbed dose distributions from plan libraries delivered through two phantoms. The major findings of this study are that the multi-phase 4D (MP4D) dose delivery approach has acceptable dosimetric quality without introducing inverse interplay effects.

The implication of this study is that MP4D delivery offers a promising new alternative approach to motion mitigation that provides good dosimetric quality with moderate technical complexity. The magnitude of technical complexity is an important characteristic because it can be a barrier on the path of translation of new technologies to clinical practice. One such technology, ion beam tracking, entails rapidly modifying planned beam spot positions to the real-time detected target motion (12, 33). Consequently, the dose distributions delivered to a patient cannot be fully confirmed by pre-treatment quality assurance testing. One type of beam tracking, called 4D-optimized tracking, that pre-computes tracking vectors to take anatomical motion from 4DCTs into account during planning, still exhibited inverse interplay effects and other dose degradations (10). The process of adjusting planned beam spot positions with tracking vectors results in cold- and hotspots in the proximal normal tissues. Our results suggest that, in the absence of respiratory-motion-related uncertainties (baseline drifts and changes to the tumor trajectory), clinically acceptable projected therapeutic outcomes could be achieved with the MP4D approach without inducing significant hotspots in normal tissues (due to inverse interplay effects) and the target volume (due to interplay effects). This study provides new evidence that, when considered with other recent studies (13, 29), suggest it may be feasible to translate the MP4D approach to clinical practice for both carbon ion and proton beam treatments. It must be emphasized that the MP4D approach is still in the early stages of preclinical development and testing; further work is needed to understand how dosimetric quality is impacted by irregular motion (*e.g.*, baseline drifts and changes to target trajectories caused by coughs and sneezes). Furthermore, additional research is needed to implement and evaluate MP4D deliveries with real-time corrective tracking and to compare the MP4D approach to the 4D-optimized tracking approach.

This work is broadly coherent with previous literature on motion mitigation approaches for proton and ion therapies. Our findings extend previous preliminary studies that suggested feasibility of a novel dose delivery system (M-DDS) with integrated motion-synchronization strategies (13, 29). The MP4D delivery approach poses a straightforward solution to solve the limitations of 3D tracking and 4D-optimized tracking. Previous research at GSI focused on 3D tracking, which required utilizing a fast wedge system to modulate beam spot delivery depth and compensate for motion-induced target depth changes in real-time, during treatment delivery (33, 34). Experiments confirmed the range compensation capabilities of this system. The 3D tracking method was reported by Saito et al. (33), and compensated for translational target motion (only), but beam spot delivery accuracy was still within 5 mm in the lateral and longitudinal directions. Importantly, this work revealed several complications with potentially important clinical implications for certain situations. First, the so-called “inverse interplay” effect was observed, due to differences in motions of the target and the tissue of the entrance channel (35). Second, complex motion, such as tumor rotations or deformations could not be fully compensated for (36). Finally, for tissues with large heterogeneities, no solution was found to compensate for motion-induced range changes. 4D-optimized tracking (12), or online adaptive tracking (37) partially solved the latter problem. However, that 4D tracking implementation encountered several obstacles, including limitations on the available hardware speed and memory, and difficulties with synchronizing the system timing. These issues rendered the system obsolete; it was dismantled and replaced with the motion-synchronized dose delivery system described here and elsewhere (13).

In consideration of the above, the MP4D delivery strategy is generally less complex, and allows for integrating a variety of treatment planning strategies, such as 4D optimization (38). It also accommodates the pre-treatment quality assurance methods similar to those currently used clinically (29). Our findings on dosimetric quality are comparable to those of the system at NIRS. At NIRS, phase-controlled rescanning is used to deliver a full set of rescans during each gating window (39). X-ray fluoroscopy detects when the tumor is within a pre-defined gating window, resulting in accurate treatment to the tumor volume. Typical results for phase-controlled rescanning were a *D*_95_ of over 95 %. Further, clinical outcome data revealed 2-year survival rates were as high as 82 % for stage 3 lung cancers treated with passive ion beams (40). This method relies on the fast-scanning magnets of the HIMAC accelerator at NIRS, and slower beam deliveries may not be able to achieve the same results. In contrast, our approach allows for continuously adapting the delivery sequence to detected motion, with minimal delivery pauses (a maximum 21% observed increase to total delivery time with regular motion), and with minimal residual motion during active beam delivery. For these reasons, it appears that the MP4D approach may find broader applicability than is possible with other approaches.

Our study has several strengths. First, we performed all of our measurements with a modular and portable dose delivery system (13), with integrated solutions for motion-synchronized dose delivery. This is potentially broadly applicable and the M-DDS has already been demonstrated at multiple centers, including CNAO and GSI. Additionally, as the motion mitigation portion of the M-DDS is an optional module, the M-DDS requires no modifications to run either with or without the motion mitigation module. This allows for implementing the M-DDS in a stepwise manner into existing facilities. We also selected methods for assessing dosimetric quality that are standard techniques within ion therapy centers (26, 41). Further, the range changes were measured by delivering beams to simple phantoms rather than anthropomorphic phantoms (42–44), eliminating additional variables, such as range uncertainties associated with variations in tissue density, irregular breathing patterns, and generally more complex range changes that are found in a human anatomy. As a result, the delivery quality more directly reflects the capabilities of the MP4D deliver approach. Nevertheless, geometries that are more complex could further confirm that our motion-synchronized dose delivery strategy can compensate for range changes and represent scenarios that are closer to clinical conditions. Further, in later stages, pre-clinical tests will be performed with anthropomorphic phantoms to characterize the full capabilities of the M-DDS.

Our study had several limitations. At the time of these experiments at GSI, our beam gating system (based on radiofrequency knockout extraction) could not yet fully gate the beam (21), and the accelerator system could not yet provide beams of multiple energies in any one delivery. We discuss both of these limitations in detail here. The inability to completely gate the beam results in an insignificant but observable amount of undesired radiation that only slightly degraded the dose distributions. Specifically, a trend was apparent (**Figure 7**) that, as the number of motion phases increased, the average absorbed dose in the CTV increased as well. The increase was under 0.4% undesired, additional dose. To overcome the limitation of having only single energies available, a passive range shifter system was utilized to modulated beam energy and range. Due to the additional material and air gaps between the range shifter plates, the beam spot size was strongly dependent on the amount of range shifter material (29). This was not a serious limitation, as the range compensation capabilities could still be demonstrated, and the TRiP4D TPS was updated to take the correct spot sizes into account in the dose calculation algorithm. In the next stage, the experiments reported here will be reproduced at CNAO, where the gating system is tuned for therapy and beam energies of 120 – 400 MeV/u are available for carbon ions (20). Another limitation of our study is that we have not tested the MP4D delivery strategy under more complex respiratory scenarios, including baseline drifts, changes to breathing amplitude and changes to breathing speed, as well as more extreme respiratory irregularities, including coughing. Some of these irregular motion scenarios could result in cold and hotspots in the target volume and dose to surrounding tissue. These capabilities will be implemented in later stages, along with improved beam gating, which will be used for handling unforeseen motion, including coughing. Finally, we did not compare the MP4D delivery strategy to other motion handling methods currently used in clinics [including the gating methods, and ITV-based deliveries with rescanning (45)] or study the results of combining motion mitigation strategies. These strategies are studied in detail elsewhere (45, 46), and delivery degradations in the complete absence of motion mitigation are shown in this study.

The results presented in this work are part of an ongoing effort to develop motion-synchronized dose delivery strategies at GSI. The motion-synchronized dose delivery system was previously assessed for safety (29), and the strategy has been validated against other approaches, including ITV-based deliveries with rescanning (45). In the future, dose degradation due to irregular motion and differences between motion during imaging and during delivery will be quantified, and corrective motion tracking will be implemented to correct for irregular target motion. The long-term goal is to translate the multi-phase 4D delivery approach and motion-synchronized dose delivery system into clinical use at CNAO.

## Conclusions

We validated the dosimetric performance of multi-phase 4D treatment delivery with scanned ion beams in the presence of multiple beam ranges. The results of this work demonstrate that it is possible to deliver motion compensated dose distributions in the presence of anatomical heterogeneities. Notably, the dosimetric performance was achieved without high technological demands or specialized equipment for mitigating target motion.

## Data Availability Statement

The raw data supporting the conclusions of this article will be made available by the authors, without undue reservation.

## Author Contributions

ML, CG, and WN wrote the manuscript. CG and MD devised the project. ML and MD developed the M-DDS that is the device validated in this work. WN and CG supervised this work. ML, MW, TS, AP, and CG performed experiments and gathered data. ML, MW, CG, and TS contributed to analyzing the data of this study, including log file reconstructions and data post-processing procedures. All authors contributed to the article and approved the submitted version.

## Funding

This project has received funding from the European Union’s Horizon 2020 research and innovation program under the Marie Skłodowska-Curie grant agreement No 675265, OMA – Optimization of Medical Accelerators, from the Charles E. Coates travel award (LSU), and from the August Family Professorship (LSU).

## Conflict of Interest

The authors declare that the research was conducted in the absence of any commercial or financial relationships that could be construed as a potential conflict of interest.

## Publisher’s Note

All claims expressed in this article are solely those of the authors and do not necessarily represent those of their affiliated organizations, or those of the publisher, the editors and the reviewers. Any product that may be evaluated in this article, or claim that may be made by its manufacturer, is not guaranteed or endorsed by the publisher.
